# The tumour is in the detail: Local phylogenetic, population and epidemiological dynamics of a transmissible cancer in Tasmanian devils

**DOI:** 10.1111/eva.13569

**Published:** 2023-06-20

**Authors:** Rodrigo Hamede, Nicholas M. Fountain‐Jones, Fernando Arce, Menna Jones, Andrew Storfer, Paul A. Hohenlohe, Hamish McCallum, Benjamin Roche, Beata Ujvari, Frédéric Thomas

**Affiliations:** ^1^ School of Natural Sciences University of Tasmania Hobart Tasmania Australia; ^2^ CANECEV, Centre de Recherches Ecologiques et Evolutives sur le Cancer Montpellier France; ^3^ School of Biological Sciences Washington State University Pullman Washington USA; ^4^ Department of Biological Sciences, Institute for Bioinformatics and Evolutionary Studies University of Idaho Moscow Idaho USA; ^5^ Centre for Planetary Health and Food Security Griffith University, Nathan Campus Nathan Queensland Australia; ^6^ CREEC, MIVEGEC (CREES) University of Montpellier, CNRS, IRD Montpelier France; ^7^ Centre for Integrative Ecology, School of Life and Environmental Sciences Deakin University Waurn Ponds Victoria Australia

**Keywords:** cancer evolution, disease ecology, host‐pathogen coevolution, phylodynamics, Tasmanian devil facial tumour disease, virulence

## Abstract

Infectious diseases are a major threat for biodiversity conservation and can exert strong influence on wildlife population dynamics. Understanding the mechanisms driving infection rates and epidemic outcomes requires empirical data on the evolutionary trajectory of pathogens and host selective processes. Phylodynamics is a robust framework to understand the interaction of pathogen evolutionary processes with epidemiological dynamics, providing a powerful tool to evaluate disease control strategies. Tasmanian devils have been threatened by a fatal transmissible cancer, devil facial tumour disease (DFTD), for more than two decades. Here we employ a phylodynamic approach using tumour mitochondrial genomes to assess the role of tumour genetic diversity in epidemiological and population dynamics in a devil population subject to 12 years of intensive monitoring, since the beginning of the epidemic outbreak. DFTD molecular clock estimates of disease introduction mirrored observed estimates in the field, and DFTD genetic diversity was positively correlated with estimates of devil population size. However, prevalence and force of infection were the lowest when devil population size and tumour genetic diversity was the highest. This could be due to either differential virulence or transmissibility in tumour lineages or the development of host defence strategies against infection. Our results support the view that evolutionary processes and epidemiological trade‐offs can drive host‐pathogen coexistence, even when disease‐induced mortality is extremely high. We highlight the importance of integrating pathogen and population evolutionary interactions to better understand long‐term epidemic dynamics and evaluating disease control strategies.

## 1 INTRODUCTION

Phylodynamics, or the study of pathogen phylogenies shaped by the interaction between epidemiological, immunological and pathogen evolutionary processes, offers a powerful framework to quantify disease dynamics across systems (Fountain‐Jones et al., 2018; Grenfell et al., 2004; Volz et al., 2021). This unified framework harnesses pathogen phylogenetic relationships and population genetics, and can be used to infer changes in transmission (e.g., Fountain‐Jones et al., 2017), estimate when and where an outbreak began (e.g., Faria et al., 2014) and assess the efficacy of disease control efforts (e.g., Dellicour et al., 2018). Phylodynamic models leverage molecular sequence data sampled from infected hosts over time to estimate relevant parameters, such as the rates of genetic change, demographic growth, and transmission between hosts, and to reconstruct the ancestral relationships of pathogens (Grenfell et al., 2004). Phylodynamic methods can also be used to provide estimates of effective pathogen population size (hereafter pathogen genetic diversity) and growth rate through time, and the host population and epidemiological characteristics that may influence this pattern (Gill et al., 2016; Volz & Didelot, 2018). For example, the genetic diversity of human immunodeficiency virus (HIV) through time was more related to the proportion of susceptible hosts that become infected over a particular time period, rather than the total proportion infected at a point in time (Gill et al., 2016). Whilst principally used to quantify virus dynamics, these techniques have been increasingly employed on the genomes of other more slowly evolving pathogens (Kao et al., 2014) such as *Mycobacterium tuberculosis* (the causative agent of tuberculosis) (Kao et al., 2016), yet rarely on larger genomes (but see Patton et al., 2020). Moreover, phylodynamic models show great promise in tracking somatic cell dynamics within and between hosts (Stadler et al., 2021).

Oncogenic processes have been increasingly acknowledged as a conservation threat (McAloose & Newton, 2009; Pesavento et al., 2018), and a growing number of directly transmissible and virus‐associated cancers have been reported in terrestrial and aquatic environments (Hamede, Owen, et al., 2020). Cancer is a ubiquitous disease that affects nearly all multicellular organisms (Domazet‐Loso & Tautz, 2010), however, in most cases tumours have limited transmission potential and are forced to adapt to a single host because their evolutionary products die within the host. In the case of directly transmissible cancers, malignant cells are able to jump from host to host, providing an evolutionary pathway for continued evolution and adaptation, even after the host succumbs to the disease. Tasmanian devils (*Sarcophilus harrisii*), a carnivorous marsupial endemic to the island state of Tasmania, have been decimated by a directly transmissible cancer, devil facial tumour disease (DFTD), for more than 25 years (Cunningham et al., 2021). The disease is a clonal tumour cell line, transmitted by direct inoculation of tumour cells when susceptible and infected individuals bite each other (Hamede et al., 2013; Pearse & Swift, 2006). DFTD was first observed in 1996 in north‐eastern Tasmanian (Hawkins et al., 2006) and since then has spread to 95% of the species' distributional range (Cunningham et al., 2021; Lazenby et al., 2018).

Phenotypic and genotypic responses to DFTD have been documented in the host, at individual and population levels, suggesting that evolutionary processes are underway (Hamede, Madsen, et al., 2020; Hohenlohe et al., 2019). Host adaptations in response to the DFTD epidemic have been diverse, including developing anti‐DFTD antibodies resulting in tumour regressions (Margres et al., 2020; Pye, Hamede, et al., 2016), variation in susceptibility to infection due to differences in expression levels of natural (IgM) versus specific (IgG) antibodies (Ujvari et al., 2016), sex‐biased differences in tolerance to infection (Ruiz‐Aravena et al., 1891) and changes in allele frequencies in genomic regions associated with cancer and immune function (Epstein et al., 2016; Fraik et al., 2020; Stahlke et al., 2021). Adaptative processes and disease dynamics in the wild are expected to be driven by both hosts and pathogens, their biology and resulting evolutionary interactions (Alizon et al., 2013; Galvani, 2003). With transmissible cancers, fitness is not restricted by the death of the host, and the transmission of clonal cancer cell lineages ensures their evolution in the long term, similar to true pathogens. Accordingly, somatic evolution in DTFD has led to several sublineages emerging during the course of the epidemic (Kwon et al., 2020; Patton et al., 2020). Competition between tumour lineages for available hosts is expected to drive the evolution of tumour traits such as transmissibility and virulence, which in turn can shape epidemic and population dynamics (Patton et al., 2020).

Often, infectious diseases are at sub‐optimally high virulence upon emergence and then evolve to a state of coexistence with their hosts over time (Langwig et al., 2015), which may be the case for DFTD as shown by ecological models (Wells et al., 2019) and a range‐wide phylodynamic study (Patton et al., 2020). Indeed, whilst DFTD is a lethal cancer that has caused dramatic population declines throughout Tasmania, differences in epidemic patterns have been observed at local scales (Hamede et al., 2012). Detailed studies of localised DFTD lineage dynamics have yet to be conducted, and a population in north‐western Tasmania (West Pencil Pine, WPP hereafter) has been systematically monitored since the beginning of the epidemic outbreak in 2006. Therein, the first evidence that tumour genetic variants could be associated with infection rates and population response was documented (Hamede et al., 2012). Relative to other devil populations, DFTD infection rates and population declines were reduced during the first 5 years after the epidemic outbreak, and these patterns were associated with the high prevalence of a tetraploid tumour karyotype, observed only at a small number of locations. Subsequently, however, a sudden replacement by a diploid karyotype (B clade, see Kwon et al., 2020) resulted in a significant increase of DFTD prevalence and population decline (Hamede et al., 1814). Although aneuploidy is common in solid tumours (Cappello et al., 2014; Kops et al., 2005), the vast majority of DFTD tumours have been diploid (Murchison et al., 2012) with tetraploidy being rare (Hamede et al., 1814; Pearse et al., 2012).

Recent phylogenetic studies support the epidemiological evidence that DFTD originated in north‐eastern Tasmania during the 1980s or 1990s with initial southward and westward spread (Patton et al., 2020). Monophyletic clades appeared early in the epizootic and progressed westward, with two predominant lineages in the WPP population (the tetraploid A2 and diploid B clades, see Kwon et al., 2020) currently overlapping in their geographic distribution (Murchison et al., 2012; Patton et al., 2020). Another tetraploid clade (Clade C) with a more limited distribution has not been sampled since 2013 in the WPP population (Kwon et al., 2020). As the epizootic unfolded, devil populations declined dramatically at regional scale (Cunningham et al., 2021; Lazenby et al., 2018), accompanied by a reduction in the effective reproduction number of the disease (Patton et al., 2020). Phylodynamic inference at local scales for DFTD has been difficult as until recently, sequence datasets had not been collated over a period sufficiently long for adequate temporal signals to be identified and evaluated (Biek et al., 2015). Analysing epidemiological data at local scales where the majority of infections are sequenced (i.e., more complete transmission chains) allows detailed understanding of transmission dynamics and provides a better temporal resolution to study pathogen evolutionary processes than by analysing sparser species‐wide patterns (Fountain‐Jones et al., 2022; Frost & Volz, 2010). The long‐term systematic and intensive monitoring effort at the WPP population provides an ideal data set with which to understand how local changes in tumour lineages can drive temporal epidemiological and host population patterns. Such fine‐scale analyses can also help to differentiate transient dynamics from long‐term epidemic characteristics. Integrating research on tumour genetic variation, population response and epidemic outcome will help in determining potential changes in tumour characteristics associated with high transmission and evaluating the prospects for devil‐DFTD coexistence.

Here we evaluate changes in local epidemiological patterns and DFTD phylodynamics at WPP, a population subject to 12 years (six devil generations) of systematic data collection. This temporal scale represents a suitable period of time to investigate changes in infection dynamics and population response (Hamede et al., 2012; Lazenby et al., 2018; McCallum et al., 2009), localised changes in disease dynamics (Hamede et al., 1814) and possible adaptations in response to the DFTD epidemic (Epstein et al., 2016; Patton et al., 2020). We estimate DFTD genetic diversity through time by applying a Bayesian phylogenetic approach (Karcher et al., 2017) to analyse complete tumour mitochondrial genomes. While mitochondrial diversity is an underestimate of tumour genetic diversity (Patton et al., 2020), the relatively high rate of mutation and lack of recombination compared to nuclear DNA regions makes the mitochondrial genome useful for phylogenetics (e.g., Brown et al., 1979). Moreover, we model how DFTD genetic diversity relates to devil population size, DFTD prevalence and force of infection through time, to understand the association of tumour genetic diversity in shaping epidemiological patterns. This approach allows inference of the timing of DFTD arrival at the study population as well as assessment of how pathogen genetic variation drives infection dynamics, population response and possible host‐pathogen evolutionary processes. We discuss the implications of host‐tumour interactions and evolutionary dynamics for the management of this and other emerging infectious diseases in wildlife populations.

## 2 METHODS

### 2.1 Study site and data collection

The study site (WPP) is a 25 km^2^ area of private forest production land in north‐western Tasmania (41°31′S, 145°46′E). The population has been systematically monitored at 3‐month intervals since the first detection of DFTD in May 2006. Tumour samples were collected between May 2006 and August 2017. The timing of surveys has been established to coincide with important devil life‐history events: February (during juvenile dispersal and prior to mating season), May (immediately after mating season), August (when females are carrying large furred young in the pouch) and November (females in late lactation and young in dens). All trapping sessions involved setting 40 traps over 10 consecutive nights within a capture‐mark‐recapture framework. Custom‐built traps, constructed from 30 cm PVC pipe, were baited with meat and checked daily after dawn. All devils were permanently marked with microchip transponders (Allflex NZ Ltd.) upon first capture. For individuals presenting with clinical signs of DFTD (one or more tumours), a photo‐identification was taken for each tumour and a unique number assigned to it. Tumour biopsies were taken using 3–6 mm sterile biopsy punches (Steifel®) depending on tumour size, and tumour tissue was immediately transferred into RNAlater solution.

#### 2.1.1 Devil population size

To estimate the population size, we fitted a time‐varying Jolly‐Seber model (POPAN) formulation (Schwarz & Arnason, 1996) using R 3.6.2 (R Core Team, 2018) package *RMark* (Laake, 2013), which acts as an R interface to capture‐recapture software MARK (White & Burnham, 1999). Our POPAN modelling approach is suitable for open populations without the assumption of closure. The model takes into account population processes such as mortality, recruitment and capture heterogeneity and is particularly suitable for long‐term studies where population closure is not met (Williams et al., 2002).

### 2.2 Molecular work

DNA was extracted using the DNeasy Blood and Tissue Kit (Qiagen). Genomic DNA was pooled at equimolar concentration, with 16–17 samples per pool, and sequenced using an Illumina HiSeq instrument with version 4 chemistry (Illumina) and 2 × 125 base pair (bp) paired end reads (Kwon et al., 2020). Illumina whole genome sequencing libraries were generated with an average insert size of 450 bp, using standard methods according to manufacturer's instructions and tagged with Sanger index tags for multiplexing. Sequence reads were aligned to the Tasmanian devil reference genome (Devil7.1) (Murchison PMID22341448) using BWA‐MEM (http://bio‐bwa.sourceforge.net/bwa.shtml) version 0.5.9‐r16+rugo with options ‘‐l 32 ‐t 6’, resulting in ~1× coverage of the nuclear genome and ~60× coverage of the mitochondrial genome (mtDNA) (Kwon et al., 2020). MtDNA single nucleotide variants (SNVs) were called using Platypus (Rimmer PMID25017105) with default settings and ‘‐‐minRead = 3’, and ‘‐‐minPosterior = 0’. We merged all variants and ran Platypus a second time across all samples. We then removed any variants with flags ‘badRead’, ‘MQ’ (mapping‐quality), or ‘QD’ (quality‐depth) and removed variants that (1) mapped within 500 bp of the start or end of the mtDNA contig, (2) had variant allele fraction (VAF) <0.2 and (3) were present with VAF >0.2 in one or more normal devils.

### 2.3 Statistical analyses

#### 2.3.1 DFTD prevalence and force of infection

We estimated prevalence for each trapping session as the proportion of animals captured on each occasion with lesions visually identified as tumours and subsequently confirmed as DFTD by molecular analysis or histopathology (Hamede et al., 2012). Force of infection (FOI) was estimated with a two‐step process. As the transition between susceptible (no clinical signs of DFTD) and infected (presentation of tumours) states occurs in continuous time, but the sampling occurs at discrete time points under imperfect detection, we fitted a Multistate Cormack‐Jolly‐Seber (CJS) model under a Hidden‐Markov process framework with two states, ‘n’ (no tumour) and ‘t’ (tumour present). Hidden‐Markov models are suitable to model time series of continuous processes that are sampled at discrete time intervals (Zucchini et al., 2017). We considered the FOI as the transition probability from having no tumour to developing tumour (Ψnt). Only three individuals regressed tumours and fully recovered from infection during the study period, however, these tumours could not be sequenced, and the individuals were not used in our analyses. Thus, we constrained the transition probability from diseased to healthy state as zero (Ψtn = 0). We included diseased state as a covariate for parameter *S* (survival) and *p* (capture probability) and allowed model parameter estimates to vary between trapping events, fitting the final set of candidate models using the R. 3.6.2 (R Core Team, 2018) package *marked* (Laake et al., 2013). We selected the best‐fitted model on the basis of second‐order AIC (Burnham & Anderson, 2002).

#### 2.3.2 Phylodynamic methods

We aligned the mitochondrial genomes using MUSCLE (Edgar, 2004) and manually checked the alignment for errors using Geneious Prime 2021.2.2. We then constructed a maximum likelihood phylogenetic tree using PhyML (Guindon et al., 2010) and selected a suitable substitution model using smart model selection (Lefort et al., 2017). We then used this tree to assess the temporal signal in our data using root‐to‐tip regression in TempEst (Rambaut et al., 2016) and more formally using the BETs (Bayesian Evaluation of Temporal Signal) procedure (Duchene, Featherstone, et al., 2020; Duchene, Lemey, et al., 2020). Bayesian phylogenetic reconstruction was performed using BEAST 1.10.4 (Suchard et al., 2018). As DFTD in this devil population was likely to have a complex demographic history (i.e., epidemic growth followed by decline as devil population size reduced) we used a Gaussian Markov random field (GMRF) tree prior using time‐aware smoothing (Minin et al., 2008). We ran three separate MCMC (Markov chain Monte Carlo) chains for 100 million generations, with trees and parameters logged every 10,000 steps. Following the BETS procedure, we evaluated phylogenies without a clock model (no sampling dates or isochronous), a strict molecular clock as well as a relaxed uncorrelated clock by calculating marginal likelihoods using generalized stepping‐stone analysis (Baele et al., 2016). Using these marginal likelihood estimates we computed Bayes factors (BFs) to compare models. We present results from the strict clock but see Table S1 for marginal likelihood estimates for each model compared. We checked convergence within and across runs and appropriate burn‐in periods in Tracer and constructed a maximum clade credibility (MCC) tree from the combined runs (excluding 20% of the trees as burn‐in).

We estimated two complementary nonparametric coalescent measures of DFTD genetic diversity through time using two R packages; *phylodyn* (Karcher et al., 2017) and *skygrowth* (Volz & Didelot, 2018). We used the *phylodyn* method to capture fluctuations in overall genetic diversity and *skygrowth* to estimate the growth rate of genetic diversity. The advantage of fitting *phylodyn* models of genetic diversity compared to methods such as *skygrid* (Gill et al., 2016) is that differences in sampling intensity are controlled for (Karcher et al., 2017). This was particularly important for our dataset as the number of tumour samples decreased as the population declined. *Phylodyn* employs INLA (integrated nested Laplace approximation) MCMC approximation to construct 95% Bayesian credible intervals on the population size estimates (Karcher et al., 2017). We performed the *phylodyn* analysis on our BEAST MCC tree using the default settings. We then calculated the Pearson correlations between DFTD genetic diversity, FOI and prevalence as well as estimated devil population size at 6 monthly intervals. Further, we examined the correlations of these variables through time by generating cross‐correlation functions (CCFs) for pairs of each time series. This CCF functions allowed us to estimate if DFTD genetic diversity, FOI or prevalence predated increases or decreases in devil population size. We also screened for relationships among DFTD prevalence, FOI and genetic diversity using CCFs. Unfortunately, we did not have enough resolution or temporal signal to add our epidemiological and population size covariates to our Bayesian coalescent model (Gill et al., 2016). We extracted the mean DFTD genetic diversity estimates every 6 months and then averaged this data to mirror the epidemiological and population size data (i.e., at a yearly scale from 2006 to 2018). The *skygrowth* model is a Bayesian autoregressive method that models growth rate as prior for genetic diversity, and has been shown to be particularly effective in capturing periods of growth and decline from pathogen genealogies (Fountain‐Jones et al., 2020; Volz & Didelot, 2018). We fitted these models using MCMC (10 million iterations) assuming that effective DFTD genetic diversity fluctuated every 6 months over the 11‐year period. See our git repository (https://github.com/nfj1380/DFTD_phylodynamics) for our phylodynamic workflow and associated data.

## 3 RESULTS

**FIGURE 1:**
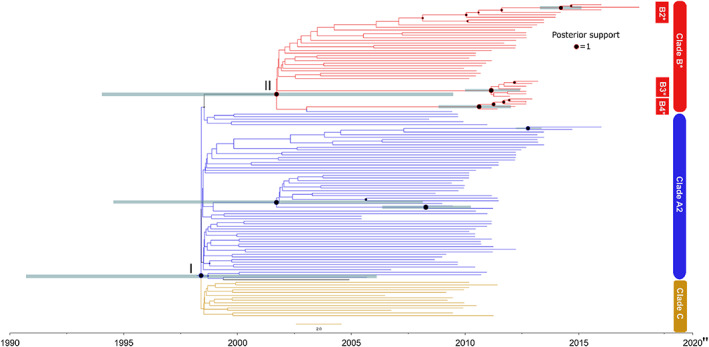
Time‐scaled phylogeny of DFTD mitochondrial sequences in the West Pencil Pine devil population. Branches are coloured based on clade designations (A2, B & C) from a Tasmania‐wide analysis (Kwon et al., 2020). Well‐supported putative sub‐clades (B2–4) are also shown. Node size reflects posterior support (see legend). *Denotes diploid tumour clade. All other clades are tetraploid. Nodes are scaled by posterior support (see legend) and grey lines indicating MRCA estimates for well‐supported nodes (posterior support >0.9). ‘I’ indicates the basal node that distinguishes Clade C from the other clades and ‘II’ indicates the node that distinguishes Clade B from A2.

Mitochondrial genomes were sequenced (16,602 base pairs, median depth of 275×) for a total of 159 tumours and aligned using the Tasmanian devil reference genome (Devil7.1) (Murchison PMID22341448). We identified 14 single nucleotide polymorphisms (SNP) loci across these genomes (see Kwon et al., 2020 for variant and genotype/haplotype information). While mitochondrial genetic variation across the population overall was low (not surprising for a clonal cancer) we found evidence of clock‐like DFTD evolution according to TempEst (slope = 1.073e^−5^, *R* ^2^ = 0.15). Our BETS analysis confirmed that there was sufficient temporal signal in the data with the strict clock model having the highest support‐based GSS likelihood estimates (log Bayes factor [BF] support: 9.85 over the model with no sampling dates, see Table S1 for marginal likelihood estimates). Using smart model selection (SMS), we found the HKY substitution model (Hasegawa et al., 1985) to have the highest support (delta BIC = 4). Based on the strict clock model, the molecular clock rate across the mitochondrial tumour genome for DFTD is 1.66e^−6^ substitutions per site per year (95% high probability density [HPD]: 3.058e^−7^–3.54e^−6^). Our most recent common ancestor (MRCA) HPD estimates for DFTD span from 1992 to 2007 (Figure 1). While there was substantial uncertainty in our time‐scaled Bayesian phylogeny, there was posterior support (posterior support >0.8) for the A2, B and C clades characterized by Kwon et al. (2020). Our analysis provides further evidence that these clades evolved concurrently, relatively early in the epizootic with the 95% HPD MRCA estimates of each clade overlapping and encompassing a period from 1993 to 2010 (I/II, Figure 1). Even though diversity was limited we did find important sub‐clade phylogenetic structure. We identified well‐supported subclades particularly within the diploid clade B (e.g., three putative mitochondrial subclades B2‐4 including between 5 and 8 tumours, Figure 1). Our 95% HPD estimates for the MRCA for these subclades included the period from ~2009 to 2015 (Figure 1). The well‐supported nodes in the B2 lineage tended just to distinguish pairs of tumours.

### 3.1 DFTD phylodynamics

**FIGURE 2:**
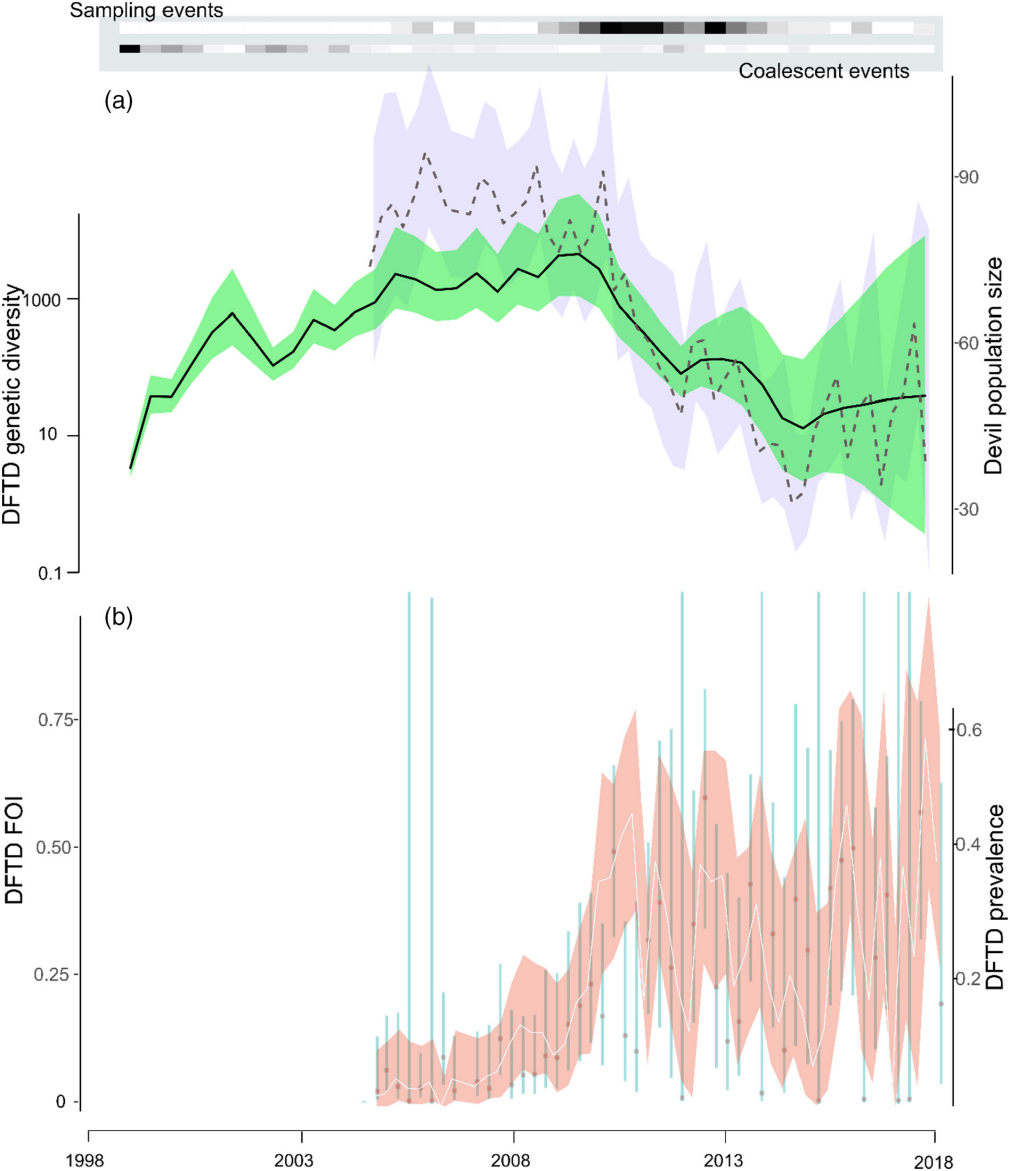
(a) Devil population size at West Pencil Pine (dotted line with purple confidence intervals) and DFTD genetic diversity (solid line with green confidence intervals). The top panel in grey illustrates the distribution of sampling and coalescent events and shows that diversity was not dependent on sampling intensity. (b) DFTD prevalence (solid line with red confidence intervals) and DFTD force of infection (FOI, vertical bars).

**FIGURE 3:**
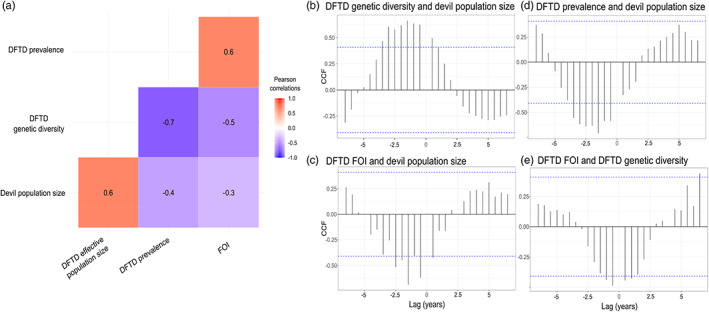
(a) Heat map showing contemporaneous Pearson correlation coefficients for the relationships between estimated DFTD genetic diversity, prevalence, force of infection (FOI) and devil population size. (b–d) Cross‐correlation functions (CCFs) for each pair of variables in relation to the mean estimated devil population. (e) CCF for the relationship between FOI and DFTD genetic diversity. The blue dotted line indicates the significance threshold (*p* = 0.05).

Our best‐fitting model for prevalence and force of infection based on AIC included variation in survival with disease state, variation in capture probability with disease state and time and variation in the transition probability from healthy to diseased with time (see Table S2). Population size remained stable during the first 6 years after disease emergence but started to decline by 2012, coinciding with an overall decrease in DFTD genetic diversity (Figure 2a). Since DFTD arrival at the study site, our *phylodyn* model revealed a gradual increase in DFTD genetic diversity plateauing in 2007–2010. After this period, DFTD genetic diversity diminished through to 2018 (Figure 2a). The trajectory of DFTD genetic diversity was positively correlated with devil population size estimates (Figure 2b, *ρ* = 0.65, Figure 3a). Our *skygrowth* model also found an increased growth rate in genetic diversity from ~2002 until ~2005 followed by a trend of decline (Figure S1). However, growth rate of genetic diversity was highest upon DFTD arrival at the study site followed by a rapid decline in growth rate until 2002 (Figure S1). Taken together, when DFTD prevalence increased, force of infection also increased and in turn devil population size decreased closely followed by a reduction in DFTD genetic diversity.

Cross‐correlation functions (CCFs) revealed that there was some evidence for lag effects as well with higher genetic diversity *phylodyn* estimates significantly correlated with higher population sizes approximately 1.5 year later (although note significant correlations between −3 years and −0.5/+0.5 years, Figure 3b). Conversely, high DFTD FOI and prevalence was correlated with low population sizes approximately 1.5 years later (Figure 3c,d). These correlations were stronger than contemporaneous estimates (Figure 3). Force of infection and prevalence were strongly positively correlated (*ρ* = 0.87, Figure 3). We detected negative correlations between DFTD genetic diversity and both FOI and prevalence (*ρ* = −0.72 and *ρ* = −0.51 respectively; Figure 3a). There was some evidence for lags between FOI and genetic diversity with high FOI correlated with low genetic diversity approximately 0.5 years later (Figure 3e).

## 4 DISCUSSION

The interplay between evolution, epidemiology and population‐scale processes is poorly understood in most wildlife diseases (Papkou et al., 2016; Penczykowski et al., 2016). Our study synthesized high‐resolution epidemiological data and population size estimates with phylodynamic methods in a localized host‐pathogen system intensively monitored since the beginning of the epidemic outbreak. We demonstrate that even after controlling for a smaller host population size sampled through time due to increasing disease‐induced mortality, the genetic diversity of the pathogen was strongly correlated with host population size. Population size of the devil could be an evolutionary ‘bottleneck’ for the tumour (i.e., low devil population size reduces the evolutionary potential of the tumour). Infectious agents, such as viruses and fungal pathogens are well known to regulate host population size and, in some cases, cause rapid selection and changes in epidemic characteristics (e.g., Savage & Zamudio, 1827). Our study allowed us to infer a similar pattern at a local scale in a transmissible cancer of clonal origin.

Devil facial tumour disease was first detected at the study site in May 2006, which at that time represented the most western point of the epidemic frontline (Hamede et al., 2012). After DFTD arrival at the study site, we observed an increase in tumour genetic diversity, which is typical at the beginning of epidemic outbreaks (Biek et al., 2007), but with growth rate declining (Figure S1). The growth rate in DFTD genetic diversity was highest at the beginning of the outbreak in this population, compared to the latter stages of our sampling period, highlighting the rapid pathogen growth in naïve host populations often observed with new epizootics. In part, the increase in tumour genetic diversity during the early stages of our sampling period was driven by the arrival of the B and C Clades from populations adjacent to our study site. However, the phylogenetic structure within Clades A2 and B that persist in the WPP population provides evidence of the importance of fine‐scale fitness landscapes shaping the genetic variation of DFTD. Our estimates of intra‐clade phylogenetic structure based on mitochondrial markers are clearly underestimates of tumour genetic diversity in the WPP. For example, in a Tasmanian‐wide study that included nuclear markers but substantially fewer individuals in the WPP population, Kwon et al. (2020) found greater structure within all three clades, with many subclades just found in the WPP population.

Regardless of the source of this genetic variation, we showed that this genetic diversity in the mitochondrial genomes is accruing in a temporally quantifiable way, which aligns with Tasmania‐wide epidemiological patterns. Our MRCA HPD estimate spans the period when DFTD likely first evolved in northern Tasmania (Patton et al., 2020). While we cannot put a precise date on the arrival of DFTD to the WPP population, it is plausible that DFTD was present at WPP several years prior to its first field detection in 2006. Our back projected phylodynamic analysis reveals a spike in growth rate ~2002, potentially associated with the formation (likely outside of the WPP area) of what would become the dominant lineage (a diploid variant, see Hamede et al., 1814) from ~2013 onwards. Between 2005 and 2009, tumour genetic diversity reached the highest point at our study site (Figure 2a), as did host population size, yet prevalence and force of infection were the lowest estimated (Figure 2b). This is opposite to what has been found in some viral‐host systems where, for example, prevalence or incidence is positively associated with HIV genetic diversity (Gill et al., 2016). When the devil–tumour interaction was in the peak of epidemic growth phase (2010–2013), DFTD genetic diversity started to decline (Figure 2), this trend is further supported by CCFs (Figure 3). This was followed by a substantial decline in devil population size and gradual levelling of prevalence and FOI, suggesting that during this timeframe various tumour lineages were weeded out, while others became better adapted and more dominant in the population. The impact of these selective sweeps on patterns of tumour genetic diversity has been observed in simulated viral genealogies under episodic positive selection over a much larger number of generations (Bedford et al., 2011). One potential mechanism driving this pattern could be differential pathogenicity or virulence amongst tumour variants. Previous studies support this hypothesis, as tetraploid tumours have a lower threshold for maximum volume than diploid tumours (Hamede et al., 2017) and low DFTD prevalence was found to be associated with the tetraploid DFTD variant (Hamede et al., 1814).

Another plausible hypothesis for the trend in low prevalence and force of infection observed whilst DFTD genetic diversity was highest, could be heterogeneities in the transmissibility of DFTD variants, which can be driven by either host or tumour mechanisms. The development of host defence strategies such as tolerance or resistance in response to pathogenic infections is fundamental for host‐pathogen evolution (Råberg et al., 2009; Read et al., 2008). A small number of devils at our study site have been observed with natural tumour regressions (Pye, Hamede, et al., 2016), particularly early in the epidemic outbreak. During our sampling period, 16 individuals were observed with regressing tumours (tumours that did not disappear but reduced their volume over 3‐ to 9‐month intervals), whilst three individuals had fully regressed tumours. It is also possible that other individuals in the population were fully resistant to infection (e.g., individuals that might have been infected but never developed tumours). Unfortunately, there is no preclinical test available for DFTD, which means animals can only be classified as symptomatic (presenting tumours) or asymptomatic (no visible tumours). Therefore, we are currently not able to test this hypothesis. Pathogen variants may also harbour genetic or phenotypic traits that are associated with differing transmissibility or infection risk (Hatherell et al., 2016; Volz et al., 2021). Successful transmission of DFTD relies on live tumour cells being transferred from host to host during injurious contact. It is possible that tumour lineages early in the epidemic outbreak were less likely to successfully establish and grow after infecting new hosts, resulting in low force of infection and prevalence, or that DFTD may accrue mutations during the incubation period (before animals are able to transmit disease) which could increase the lag between genetic diversity, prevalence and FOI. Finally, we cannot discount the possibility that the epidemic growth became exponential when a threshold number of devils were infected.

The Tasmanian devil has been affected by DFTD for more than 25 years. No local extinctions have been so far observed, despite severe population declines in most populations 3–4 years after disease arrival and early models that predicted DFTD‐induced extinction (McCallum et al., 2009). Furthermore, a recent phylogenetic study (Patton et al., 2020) sequenced the genome of 50 tumours across the distributional range of DFTD between 2003 and 2018 and found a significant decrease in the basic reproductive number of DFTD over time, suggesting that this transmissible cancer is shifting to an endemic disease. Our study population at WPP has been systematically surveyed since the epidemic outbreak in 2006 and provides a valuable illustration of how localized changes in pathogen lineages can lead to shifts in epidemic patterns, allowing the coexistence of devils and a transmissible cancer. Our results are particularly relevant to understanding the evolutionary trajectory of clonal cancer cell lines and their effects on population dynamics, and to evaluate possible parallels with the second and independently evolved transmissible cancer affecting Tasmanian devils, devil facial tumour 2 (DFT2) (Pye, Pemberton, et al., 2016). DFT2 was first reported in 2014, in southeastern Tasmania at the d'Entrecasteaux peninsula (James et al., 2019). DFT2 is likely to be of similar origin than DFTD (Stammnitz et al., 2018), however, the molecular evolution of DFT2 and its epidemiological patterns are poorly understood (but see Stammnitz et al., 2023). Similar studies able to quantify the evolutionary interactions between devil populations and DFT2, and the effects of lineage dynamics in population processes will help to predict temporal and spatial patterns of infection and determine the conservation threat this new transmissible cancer poses for the species. For example, our phylodynamic approach can be used to track down lineage diversification of DFT2 in real time, and its effects on infection rates, virulence and population dynamics. DFT2 is no longer restricted to the geographic peninsula where it originated and is spreading north where it will be subject to selective pressures imposed by DFTD. Therefore, predicting the phylodynamic interactions of these two transmissible cancers should become a research priority for the conservation of the species.

Evolutionary processes in the tumour at our study site coincide spatially and temporally with selection by DFTD on hosts. Epstein et al. (2016) found changes in host allele frequencies in genes associated with cancer and immune function in as little as 6–8 years after disease arrival, which are concurrent with changes in tumour lineages at our study site. These patterns highlight the importance of considering the adaptive potential of host‐pathogen systems and the need to evaluate the suitability of intervention strategies and conservation policies in the face of epidemic outbreaks. Eradication of DFTD is not feasible in the foreseeable future, therefore, future research should focus on further understanding functional genetic changes in the devil in response to DFTD and managing the adaptive potential of the host in light of long‐term epidemiological and selective processes (Hamede, Madsen, et al., 2020; Hohenlohe et al., 2019). Phylodynamic approaches such as ours, capable of linking genetic changes in host‐pathogen systems and their epidemic outcomes, can be further used to evaluate the biological and ecological conditions affecting transmission rate, spatial spread or disease fade out in local areas. This information can be harnessed to determine the suitability of disease control strategies, such as selective culling, stamping out or field immunizations. In the case of Tasmanian devils and DFTD, our data suggest that at a local scale, devil‐tumour selective processes are sufficient to sustain populations, with the potential to become locally adapted.

Host–parasite dynamics can be viewed as a classic example of an evolutionary arms race, where host populations evolve defence strategies against pathogens and in turn pathogens evolve mechanisms to overcome host defences and maximize transmission (Ewald, 1995). Phylodynamic approaches are now playing an important role in illuminating the evolutionary history of pathogens as well as assessing the temporal and spatial patterns of infection (Byrne et al., 2019; Grenfell et al., 2004; Nelson & Holmes, 2007). Our study provides a broader evolutionary and epidemiological understanding of one of the most lethal infectious diseases recently documented, linking tumour genetic diversity to population dynamics. The phylodynamic approach employed herein, combined with robust field‐based epidemiological data, allowed us to differentiate transient and long‐term epidemic cycles and to track periods of low and high infection rates with contemporary changes in pathogen genetic diversity. Our results support the view that selective processes can drive the coexistence of host populations and pathogens (Wells et al., 2019), even when disease‐induced mortality is extremely high. We cannot discount, however, the possibility that future changes in tumour lineages, and other non‐tumour‐related threats such as habitat reduction/fragmentation, road kill and genetic deterioration in local populations, could alter disease dynamics and the long‐term viability of devil populations at a local scale.

We acknowledge that our findings are limited by the low levels of variation we detected in the mitochondrial genomes. Whilst we did detect clock‐like evolution in the relatively large numbers of mitochondrial genomes we screened, future population‐specific studies capturing variation across the tumour genome (e.g., Patton et al., 2020) will refine our estimates of tumour genetic diversity across time. For example, having higher genomic resolution would likely increase the accuracy and precision of our estimates for when the diploid clade arrived in the population. Moreover, sampling genetic variation across the genome would also allow us assessing if the evolutionary pressure on the tumour was decreasing or increasing across time (e.g., using the RELAX framework; Wertheim et al., 2015). Matching high‐resolution tumour diversity estimates with equally high‐resolution estimates of host genetic diversity (e.g., Hendricks et al., 2017) promises novel insights into the evolutionary arms race between devils and tumours.

Our study emphasizes the importance of integrating cross‐disciplinary approaches capable of tracking pathogen and population evolutionary processes to understand disease dynamics. We are facing a century characterized by an increasing number of emerging infectious diseases (Preece et al., 2017), which is a major concern for biodiversity loss, wildlife and human health (Cunningham et al., 2017). Complete eradication of infectious diseases in wildlife is extremely rare, which highlights the need for integration of genomics with population dynamics studies to help predict long‐term epidemiological patterns and evaluate the suitability of alternative disease control strategies.

## CONFLICT OF INTEREST STATEMENT

The authros declare no competing interest.

## Supporting information


Appendix S1

## Data Availability

The data supporting this research is archived on the Dryad Digital Repository: https://doi.org/10.5061/dryad.s1rn8pkdd. Code files and sequence alignment used in this study are also available on GitHub: https://github.com/nfj1380/DFTD_phylodynamics.
