# Supplementary material for: The tumour is in the detail: Local phylogenetic, population and epidemiological dynamics of a transmissible cancer in Tasmanian devils
Source: Evol Appl. 2023 Jun 20;16(7):1316–27. doi: 10.1111/eva.13569 (PMC10363845; doi:10.1111/eva.13569)
Supplement: Supplementary file 1 — Appendix S1 [file EVA-16-1316-s001.docx]

**The tumour is in the detail: local phylogenetic, population and epidemiological dynamics of a transmissible cancer in Tasmanian devils.**

**Supplementary files**


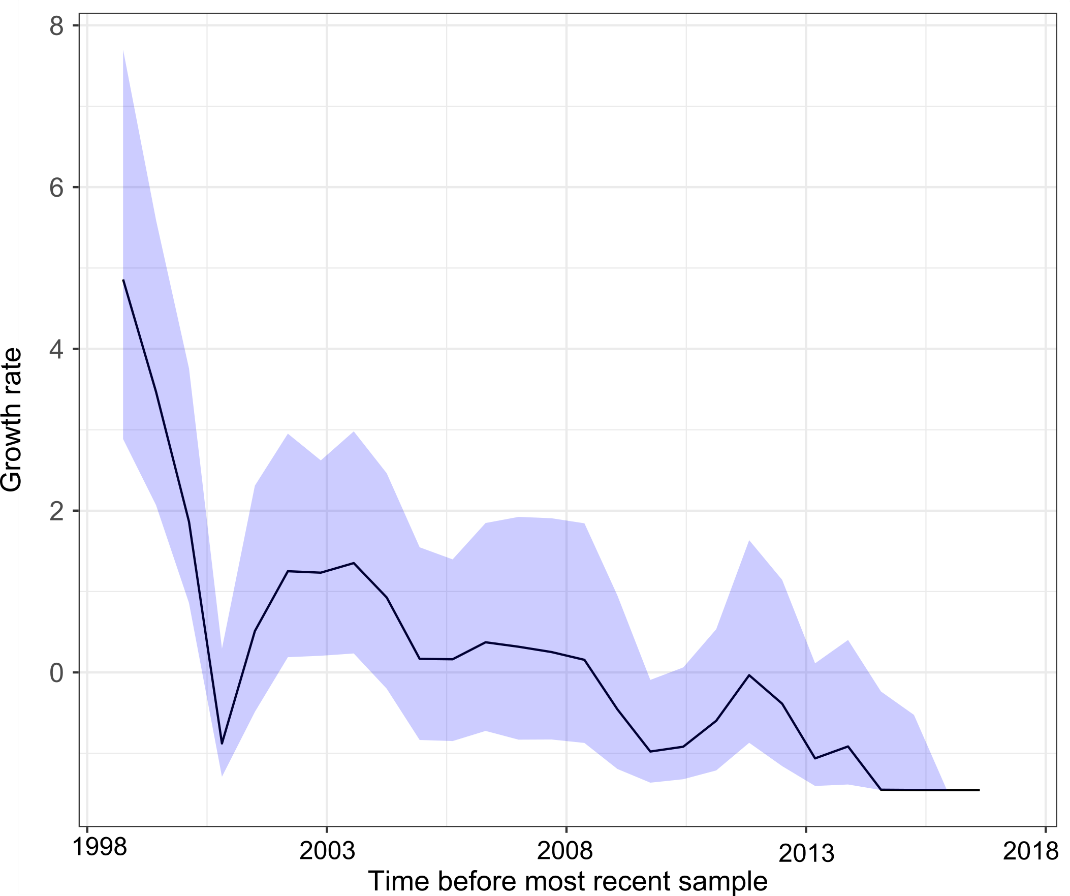


**Fig. S1:** *Skygrowth* plot showing the declining growth rate of genetic diversity through time. Blue shading indicates 95% highest posterior density (HPD) estimates.

**Table S1:** Marginal-likelihood estimates for the 3 candidate molecular clock models inferred using generalized stepping stone (GSS) samplers.

| Clock prior |  | GSS |  |
| --- | --- | --- | --- |
|  | run 1 | run 2 | average |
| Strict clock (no dates) | -22506.7 | -22507.0 | -22506.85 |
| Strict clock (with dates) | -22497.62 | -22496.38 | -22497.00 |
| Relaxed clock (no dates) | -22510.167 | -22510.32 | -22510.244 |
| Relaxed clock (with dates) | -22500.566 | -22500.867 | -22500.717 |

**Table S2:** Set of the top three ranked candidate models for the analysis of force of infection. Colum *k* refers to the number of parameters in each model and NLL refers to the negative log-likelihood. Disease state has two values, healthy and diseased. No tumour : yes tumour refers to the probability for an individual with no tumours in a given trapping session to be trapped in the next session with tumours. The code “^~^1” denote absence of covariates in the parameters for these models.

| **Competing models** | ***k*** | ***AICc*** | **ΔAICc** | ***w_i_*** | ***2* NLL*** |
| --- | --- | --- | --- | --- | --- |
|  |  |  |  |  |  |
| S(^~^disease state) p(^~^disease state + time)  Psi(^~^1 + no tumour : yes tumours + time) | 103 | 5711.37 | 0 | 0.995 | 5505.37 |
| S(^~^disease state + time) p(^~^disease state + time)  Psi(^~^1 + no tumour : yes tumours + time) | 151 | 5750.89 | 39.52 | 0.003 | 5448.89 |
| S(^~^disease state * time) p(^~^disease state + time)  Psi(^~^1 + no tumour : yes tumours + time) | 199 | 5773.09 | 61.72 | 0.001 | 5375.06 |
|  |  |  |  |  |  |

*S = survival; p = capture probability; Psi = transition probability from healthy to diseased.*
